# Enhancing Patient Engagement and Positive Step-Down Discharges Through Co-Production: A Quality Improvement Initiative in an In-Patient Rehabilitation Psychiatric Unit

**DOI:** 10.1192/j.eurpsy.2025.1638

**Published:** 2025-08-26

**Authors:** H. Tahseen, D. A. Hassoulas, K. Umla-Runge

**Affiliations:** 1 Cardiff University, Cardiff, United Kingdom

## Abstract

**Introduction:**

This Quality Improvement (QI) programme aimed to integrate co-production principles into rehabilitation psychiatry to enhance patient-centred care and facilitate positive step-down discharges. The initiative was developed in response to suboptimal audit results, revealing low patient attendance and limited positive discharges within an in-patient psychiatric unit. Recognising the critical role of rehabilitation psychiatry in supporting recovery and reintegration, the programme sought to transform patient engagement through equitable partnerships between patients and healthcare professionals.

**Objectives:**

The programme’s primary objectives were to:

1. Implement and evaluate co-production within the Care Programme Approach (CPA).
2. Increase patient attendance at CPA meetings and improve positive step-down discharges.
3. Enhance engagement, communication, and shared decision-making to achieve better patient outcomes, including reduced anxiety.

**Methods:**

A phased approach was employed, encompassing diagnostic, problem-solving, and evaluation stages. Root cause analyses were conducted using fishbone cause-and-effect diagrams and the 5-Why Technique. The Model of Improvement guided the programme, with change ideas developed and refined through Plan-Do-Study-Act (PDSA) cycles. Interventions included distributing patient information leaflets, staff training sessions, and introducing a structured CPA agenda template. Quantitative analysis using paired t-tests evaluated changes in attendance, discharge rates, and Hamilton Anxiety Rating Scale (HAM-A) scores. Qualitative data were gathered from a co-produced CPA questionnaire, with emerging themes integrated into the project’s evolution through narrative synthesis.

**Results:**

The implementation of co-production yielded significant improvements in patient engagement and discharge outcomes, resulting in a 50% increase in CPA meeting attendance and a 70% positive step-down discharge rate. Interventions were associated with reduced anxiety levels, evidenced by improvements in HAM-A scores. Qualitative analysis highlighted key themes, including challenges during community transitions, empowerment through shared decision-making, and enhanced communication with healthcare professionals. The structured CPA agenda template further improved patient-centred communication and care experiences.

**Conclusions:**

The integration of co-production within rehabilitation psychiatry fosters transformative partnerships that enhance patient engagement and clinical outcomes. This QI programme demonstrates the efficacy of patient-centred interventions, supported by structured communication tools, in empowering individuals, reducing anxiety, and improving transitions to community care. Co-production provides a robust framework for advancing rehabilitation psychiatry and optimising patient care pathways.

**Disclosure of Interest:**

None Declared

